# Synthesis and structures of dinuclear palladium complexes with 1,3-benzimidazolidine-2-thione and 1,3-imidazoline-2-thione

**DOI:** 10.1107/S2056989023000166

**Published:** 2023-01-10

**Authors:** Tarlok S. Lobana, Bandana Thakur, Rajni Aggarwal, Ray J. Butcher, Matthias Zeller, Jerry P. Jasinski

**Affiliations:** a Department of Chemistry, Guru Nanak Dev University, Amritsar 143 005, India; b Department of Chemistry, Howard University, 525 College Street NW, Washington DC 20059, USA; c Department of Chemistry X-ray Crystallography, Purdue University, Wetherill 101B 560 Oval Drive, West Lafayette, IN 47907-2084, USA; d Department of Chemistry, Keene State College, Keene NH 03435-2001, USA; Universidade de Sâo Paulo, Brazil

**Keywords:** crystal structure, dinuclear palladium complexes, benzimidazolidine- 2-thione complexes, imidazoline-2-thione complexes

## Abstract

The synthesis and structures of dinuclear palladium complexes with 1,3-benz­imidazolidine-2-thione (bzimtH) and 1,3-imidazoline-2-thione (imtH) are reported, namely, bis­(μ-1*H*-benzimidazole-2-thiol­ato)-κ^2^ *N* ^3^:*S*;κ^2^ *S*:*N* ^3^-bis­[cyanido(tri­phenyl­phosphine-κ*P*)palladium(II)], [Pd_2_(C_7_H_5_N_2_S)_2_(CN)_2_(C_18_H_15_P)_2_] or [Pd_2_(μ-*N*,*S*-bzimtH)_2_(CN)_2_(PPh_3_)_2_] (**1**), and bis­(μ-1*H*-imidazole-2-thiol­ato)-κ^2^ *N* ^3^:*S*;κ^2^ *S*:*N* ^3^-bis­[cyanido(tri­phenyl­phosphine-κ*P*)palladium(II)] aceto­nitrile 0.58-solvate, [Pd_2_(C_3_H_3_N_2_S)_2_(CN)_2_(C_18_H_15_P)_2_]·0.58C_2_H_3_N or [Pd_2_(μ-*N*,*S*-imtH)_2_(CN)_2_(PPh_3_)_2_]·0.58C_2_H_3_N (**2**). The compound [Pd_2_(μ-*N*,*S*-bzimtH)_2_(CN)_2_(PPh_3_)_2_] is located on a crystallographic twofold axis while [Pd_2_(μ-*N*,*S*-imtH)_2_(CN)_2_(PPh_3_)_2_]. 0.58(C_2_H_3_N) contains two partially occupied aceto­nitrile solvent mol­ecules with occupancies of 0.25 and 0.33. In both of these compounds, the anionic bzimtH^−^ and imtH^−^ ligands coordinate through N,S-donor atoms in a bridging mode, covering four coordination sites of two metal centers and other two sites are occupied by two PPh_3_ ligand mol­ecules. Finally, the remaining two sites of two metal centers are occupied by cyano groups, abstracted by the metals from the solvent during reaction. In the packing of the 1,3-benzimidazolidine- 2-thione and 1,3-imidazoline-2-thione complexes, there are intra­molecular π–π inter­actions involving the thione moiety as well as an N—H⋯N hydrogen bond linking the thione and cyano ligands. In addition, in **2**, as well as the π–π inter­action involving the thione moieties, there is an additional π–π inter­action involving one of the thione moieties and an adjacent phenyl ring from the tri­phenyl­phosphine ligand. There are also C—H⋯N inter­actions between the imidazoline rings and the aceto­nitrile N atoms.

## Abstract 2

The synthesis and structures of dinuclear palladium complexes with 1,3-benzimidazolidine-2-thione and 1,3-imidazoline-2-thione are reported.

## 1. Chemical context

The coordination chemistry of N,S-donor heterocyclic-2-thione ligands has been in focus for the past four decades, describing synthetic methods, bonding and structures of metal complexes (Raper, 1985 ▸, 1994 ▸, 1996 ▸, 1997 ▸; García-Vázquez *et al.*, 1999 ▸; Akrivos, 2001 ▸), analytical chemistry (Koch, 2001 ▸), charge-transfer complexes (Serpe *et al.*, 2008 ▸) and anion receptors (Bondy & Loeb, 2003 ▸). A recent survey revealed that the reactions of heterocyclic-2-thio­nes with group 10–12 metals (Ni–Pt, Cu–Au, Zn–Hg; Lobana, 2021 ▸) have led not only to the formation of a variety coordination compounds, but have also displayed other aspects of chemical reactivity. For instance, some reactions of heterocyclic thio­nes involved copper-mediated activation, and rupture of C—S (thione) bonds followed by their transformations to other forms of thio-ligands, bonded to the copper metal. Further, there has been an upsurge in explorations of the bio-activity and bio-safe potential of coordination compounds, as anti­microbial and anti­cancer agents (Lobana, 2021 ▸). 
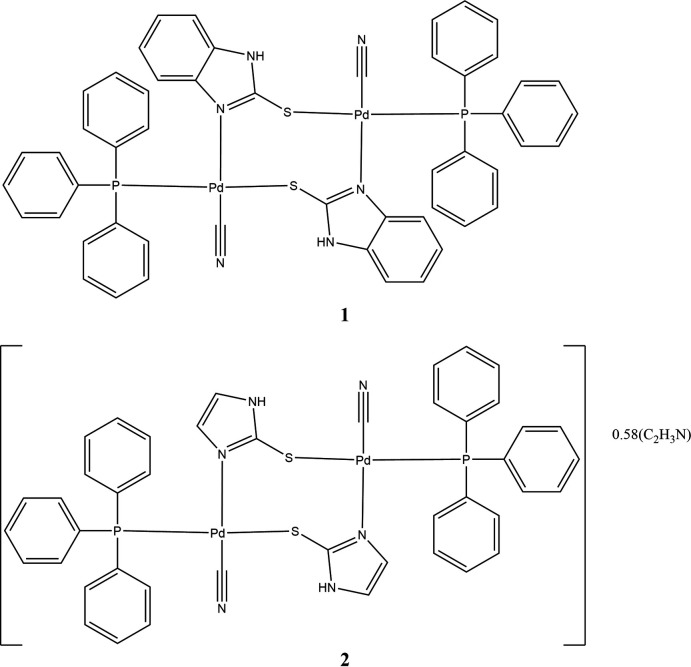


The chemistry of palladium is inter­esting because of the coordination flexibility and catalytic role of this metal in several reactions (Kostas & Steele, 2020 ▸; Lobana, 2021 ▸). In the literature, pyridine-2-thione (pytH) with palladium(II) has been reported to form dinuclear complexes, namely, [Pd_2_(μ-*N*,*S*-pyt)_4_] (Umakoshi *et al.*, 1990 ▸), [Pd_2_(μ-*N*,*S*-pyt)(μ-*S*-pyt)(κ^1^:*S*-pyt)_2_(μ-*P*,*P*-dppm)] and [Pd_2_(μ-κ^2^:*N*,*S*-pyt)_3_(κ^2^:*P*,*P*-dppm)]Cl (Mendía *et al.*, 2006 ▸), [Pd_2_Cl_2_(μ-*N*,*S*-pyt)_2_(PMe_3_)_2_] (Yamamoto *et al.*, 1991 ▸), [Pd_2_Cl_2_(μ-*N*,*S*-pymt)_2_(PMe_3_)_2_] (Yap & Jensen, 1992 ▸). Benz-1,3-imidazoline-2-thioe (bzimtH_2_) has formed one dimer, [Pd^II^_2_(μ-κ^2^:*N*,*S*-bzimt)_2_(κ^1^-*S*-bzimt)(PPh_3_)_3_]Cl·2H_2_O (Lobana *et al.* 2017 ▸). In this manuscript, some reactions of this metal with a few heterocyclic-2-thione ligands (bzimtH_2_ and imtH_2_) are described.

## 2. Structural commentary

The reaction of PdCl_2_(PPh_3_)_2_ with bzimtH_2_ in a 1:2 molar ratio in the presence of Et_3_N base was designed to form [Pd(κ^1^ *S*-bzimtH)_2_(PPh_3_)_2_] after removal of both halogens as [Et_3_NH]^+^Cl^−^. However, the X-ray crystal structure of the product revealed the formation of the unexpected dinuclear compound [Pd_2_(μ-N,S-bzimtH)_2_(CN)_2_(PPh_3_)_2_] (**1**). Another thio-ligand, imtH_2_ yielded a similar dinuclear compound, [Pd_2_(μ-*N*,*S*-imtH)_2_-(CN)_2_(PPh_3_)_2_] (**2**). In both these compounds, the anionic bzimtH^−^ and imtH^−^ ligands coordinate through N,S donor atoms in a bridging mode, covering four coordination sites of two metal centers, and other two sites are occupied by two PPh_3_ ligand mol­ecules. Finally, the remaining two sites of two metal centers are occupied by cyano groups, abstracted by the metals from the solvent during reaction.

**Figure 1:**
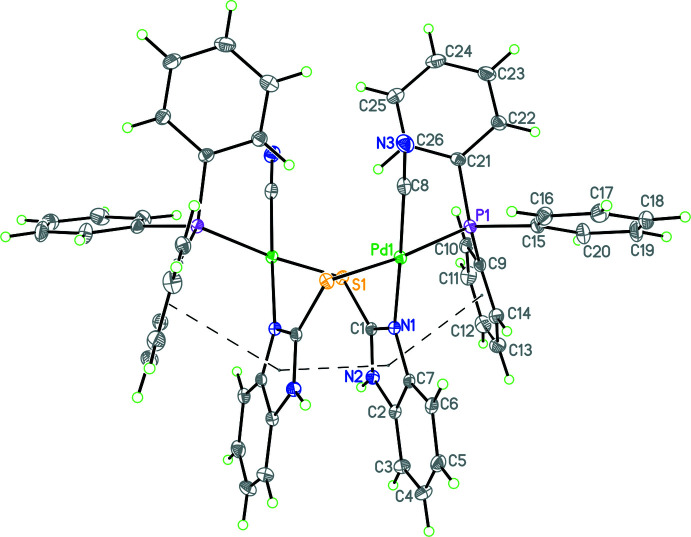
Diagram of **1** showing the atom labeling for unique atoms (the mol­ecule lies of a twofold axis; symmetry operation to generate the rest of the mol­ecule is −*x*, *y*, 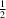 − *z*) and the strong intra­molecular π–π inter­actions involving both the thione rings and adjacent phenyl rings from the tri­phenyl­phosphine ligand. Atomic displacement parameters are at the 30% probability level.

**Figure 2:**
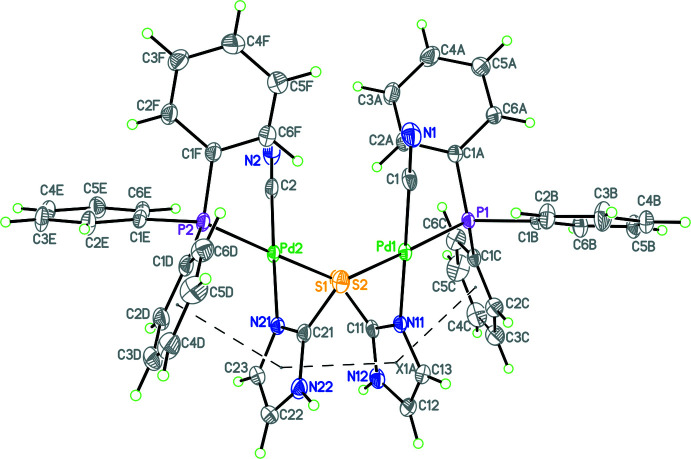
Diagram of **2** showing the atom labeling and the strong intra­molecular π–π inter­actions involving both the thione rings and adjacent phenyl rings from the tri­phenyl­phosphine ligand. Atomic displacement parameters are at the 30% probability level.

Compound **1** crystallizes in the monoclinic space group *C*2/*c*, and compound **2** in the monoclinic space group, *P*2_1_/c. Selected bond distances and bond angles are given in Tables 1 ▸ and 2 ▸, respectively. The mol­ecular structure of compound **1** is shown in Fig. 1 ▸, while that of compound **2** is shown in Fig. 2 ▸ (leaving out the aceto­nitrile solvent mol­ecules). Considering first the structure of compound **1**, here only half of the mol­ecule is unique as the mol­ecule lies on a crystallographic twofold axis. In **1**, the Pd metal atom is bonded to one P, one S, one N and one C atoms with the respective bond distances as follows: Pd—P = 2.2861 (6), Pd—S = 2.3547 (6), Pd—N = 2.0545 (17), and Pd—C = 1.959 (2) Å. The *trans* bond angles, P—Pd—S and N—Pd—C, of 172.26 (2) and 178.31 (8)°, as well as the *cis* bond angles in the range 84.93 (6)–94.24 (5)°, reveal the distorted square-planar geometry of each metal center. One of the major factors in the conformation adopted by the mol­ecule is the strong π–π inter­action between the thione moieties [*Cg*⋯*Cg*, 3.1905 (12) Å], as seen in Fig. 1 ▸. In addition, there is also a π–π inter­action between the thione moieties and an adjacent phenyl ring from the tri­phenyl­phosphine ligand [*Cg*⋯*Cg* = 3.3560 (9) Å with a slippage of 1.408 Å].

The coordination pattern of compound **2** is similar to that of **1**. Nevertheless, there are minor differences in the bond distances and angles pertaining to the two metal centers of compound **2** (Fig. 2 ▸). Thus, the respective Pd—P, Pd—S, Pd—N and Pd—C bond distances of **2** are 2.2914 (5), 2.3541 (5), 2.0345 (17) and 1.957 (2) Å (Pd1 metal center), and 2.2984 (5), 2.3542 (5), 2.0345 (17) and 1.943 (2) Å (Pd2 metal center). For both metal centers, the *trans* bond angles [P—Pd—S and N—Pd—C = 173.86 (2)–179.31 (8)°] and the adjacent bond angles [86.54 (7)– 92.80 (4)°] are similar to those of compound **1**. These bond angles again reveal the distorted square-planar geometry of each metal center of compound **2**. The various bond distances described above are normal and none unusual. Compound **1** has carbon–sulfur (C—S) bond distance of 1.728 (2), while in compound **2** it is 1.734 (2) Å. These distances are in between single (1.81 Å) and double-bond (1.68 Å) C—S distances (Huheey *et al.*, 1993 ▸). It shows a weakening of the C—S bond as a result of S to Pd coordination. The C≡N distance of the coordinated cyano group is 1.127 (3) in compound **1** and 1.143 (3) /1.148 (3) Å in compound **2**. These distances are less than the expected C=N double bond (1.28 Å) and are close to the C≡N triple bond distance (1.15 Å; Huheey *et al.*, 1993 ▸). The structure of **2** contains partially occupied aceto­nitrile solvent mol­ecules with occupancies of 0.33 and 0.25. As in the case of **1**, in **2** one of the major factors in the conformation adopted by the mol­ecule is the strong π–π inter­action between the thione moieties [*Cg*⋯*Cg* = 3.3559 (12) Å], as seen in Fig. 2 ▸. In addition, there is also a π–π inter­action between each of the thione moieties and an adjacent phenyl ring from the tri­phenyl­phosphine ligand [*Cg*⋯*Cg* distances of 3.3065 (8) Å and 3.3218 (8), respectively, with a slippage for the latter of 1.154 Å].

The IR spectrum of the bzimtH_2_ ligand showed a ν(N—H) band at 3113 (*m*), and in compound **1**, this band appeared at a lower energy, 3055 (*m*) cm^−1^. The ligand showed a diagnostic ν(C=S) band at 1179 cm^−1^, which shifted to ν(C=S), 1033(*s*) cm^−1^, owing to the change of neutral bzimtH_2_ ligand to the bzimtH^−^ anionic form, coordinating through N,S donor atoms. The PPh_3_ ligand showed its characteristic ν(P—C_Ph_) band at 1097(*s*) cm^−1^ in compound **1**. A band at 1734 cm^−1^ was assigned to the coordinated cyano group. The IR spectroscopic bands of compound **2** are similarly assigned: ν(N—H), 3050 (*m*), ν(C=S), 1020 (*m*), ν(P—C_Ph_), 1105 (*s*) and ν(C≡N), 1740(*s*) cm^−1^.

In conclusion, the chemistry of heterocyclic-2-thio­nes remains enigmatic, probably due to the angular flexibility at sulfur, and also due to the short bite angle of the N,S-donor set in case it chelates with the formation of four-membered rings. This leads to a greater tendency of these thio-ligands in anionic forms to adopt bridging modes, noted as for example in dinuclear complexes (Raper, 1997 ▸; Lobana, 2021 ▸). Benz-1,3-imidazoline-2-thione (bzimtH_2_) has formed an N,S-bonded symmetrically bridged dinuclear compound, and so is the case with 1,3-imidazolidine-2-thione, and these are analogous to literature reports (Yamamoto *et al.*, 1991 ▸; Yap & Jensen, 1992 ▸).

## 3. Supra­molecular features

**Figure 3:**
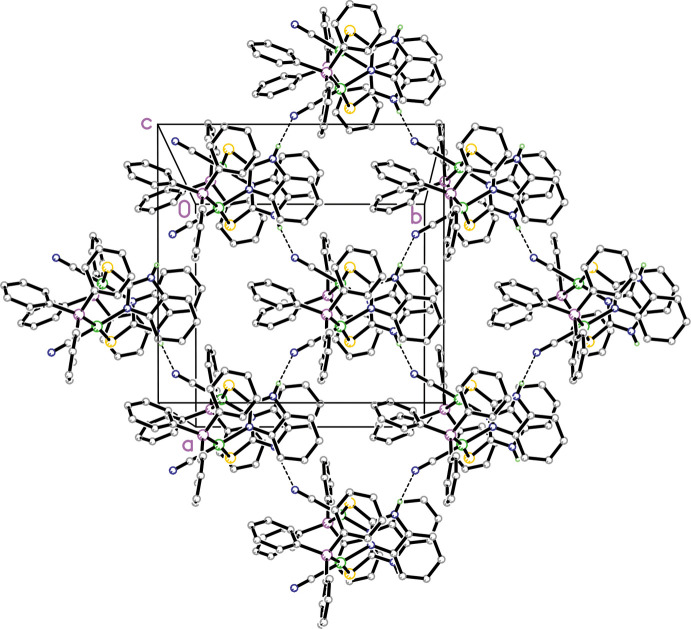
Diagram showing the packing for **1** showing the two inter­molecular 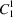 (7) N—H⋯N hydrogen-bonded chains in the [110] and [ 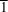 10] directions. Hydrogen atoms not involved in hydrogen bonding are omitted for clarity. Symmetry operation to generate the rest of the mol­ecule is −*x*, *y*, 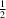 − *z*.

**Figure 5:**
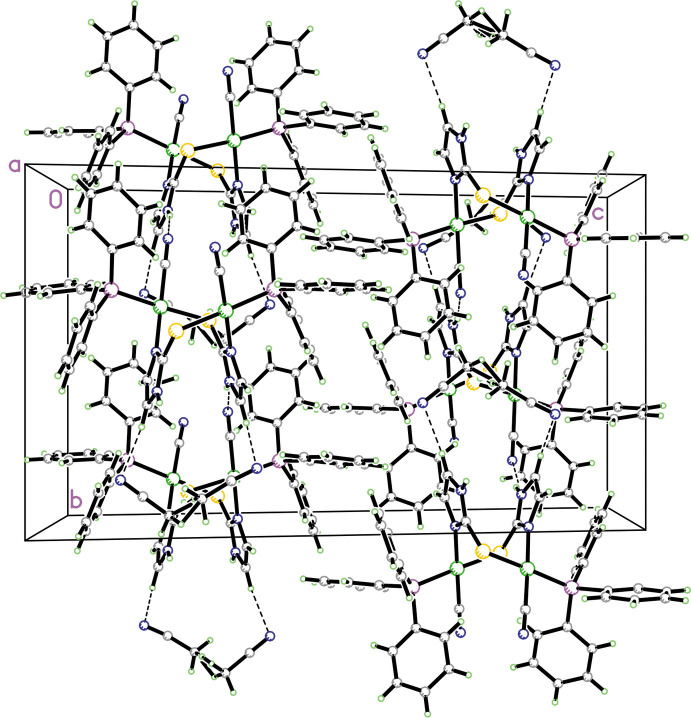
Diagram for **2** showing the packing viewed along the *a-*axis direction. N—H⋯N hydrogen bonds and C—H⋯N inter­actions involving the aceto­nitrile mol­ecules are shown as dashed lines.

In the packing of **1** and **2** there are similar trends in both hydrogen-bond patterns and intra­molecular inter­actions. In both structures, there are strong intra­molecular π–π inter­actions involving the thione moiety and adjacent phenyl rings from the tri­phenyl­phosphine ligand as discussed above. Both **1** and **2** have a similar hydrogen-bonding pattern (numerical details in Tables 3 ▸ and 4 ▸), as shown in Figs. 3 ▸ and 4 ▸. In each, the N—H group of the thione moiety forms an inter­molecular hydrogen bond with an adjacent N atom from the coordinated cyanide anion and these form 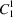 (7) chains (Etter *et al.*, 1990 ▸) in the [110] and [ 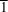 10] directions. In addition, in **2** there are also C—H⋯N inter­actions between the imidazoline rings and the partially occupied aceto­nitrile N atoms and this is shown in Fig. 5 ▸.

## 4. Database survey

A search of the Cambridge Structural Database for complexes of palladium with either 1, 3-benzimidazolidine- 2-thione or 1,3-imidazoline-2-thione returned nine hits for the former (BEYRUV and BEYWAG, Sandhu *et al.*, 2018 ▸; PONKOT, PONKUZ, PONLAG, PONLEK and PONLIO, Talismanova *et al.*, 2008 ▸; SANMOK, Talismanova *et al.*, 2004 ▸; SAQPEI, Lobana *et al.*, 2017 ▸) and three hits for the latter (APIYII, Ahmad *et al.*, 2010 ▸; BEYVUZ, Sandhu *et al.*, 2018 ▸; HAWYEJ, Kahn *et al.*, 1993 ▸, SAQPIM, Lobana *et al.*, 2017 ▸).

## 5. Synthesis and crystallization

The starting materials, namely palladium(II) chloride, tri­phenyl­phosphine (PPh_3_), 1,3-benzimidazoline-2-thione (bzimtH_2_), 1,3-imidazoline-2-thione (imtH_2_), and tri­ethyl­amine were procured from Aldrich. The solvents (aceto­nitrile, ethanol, methanol and di­chloro­methane) were of HPLC grade and were stored over mol­ecular sieves. The precursor, PdCl_2_(PPh_3_)_2_, was prepared by a literature procedure (Steffen & Palenik, 1976 ▸). The melting points were determined with a Gallenkamp electrically heated apparatus using the dried samples in capillary tubes. The analysis for carbon, hydrogen and nitro­gen were performed by using CHNS-O analyzer Flash- EA-1112 series. The IR spectra of the compounds were recorded on FTIR–SHIMADZU 8400 Fourier transform spectrophotometer in the range of 4000–400 cm^−1^ using KBr pellets.

*Preparation of the precursor, [PdCl_2_(PPh_3_)_2_]*

Palladium(II) chloride (0.050 g, 0.282 mmol) was dissolved in hot aceto­nitrile (25 mL) in a 50 mL round-bottom flask, and to it was added tri­phenyl­phosphine (0.148 g, 0.564 mol). The contents were refluxed for 1 h and the yellow complex formed was filtered and dried *in vacuo*, m.p. 551-553 K

*Preparation of **1***

To a solution of PdCl_2_(PPh_3_)_2_ (0.030 g, 0.043 mmol) in 10 mL of CH_3_CN, was added solid bzimtH_2_ (0.013 g, 0.086 mmol) followed by the addition of Et_3_N base (0.5 mL). The solution became yellowish orange and was refluxed for 6 h. The orange compound was formed on refluxing. It was separated and dissolved in a solution of methanol (4 mL) and di­chloro­methane (1 mL) in a culture tube. A slow evaporation of the reaction mixture over a period of one month, resulted in the formation of orange crystals of compound **1**. Yield: 0.015 g; 65%; m.p. 511–513 K. Analysis found: C, 57.71; H, 3.84; N, 7.50; C_52_H_40_N_6_P_2_Pd_2_S_2_ (1087.8) requires: C, 57.40; H, 3.70; N, 7.72%. IR Data (KBr, cm^−1^): ν(N—H), 3055 (*m*); ν(C–H), 2950 (*m*), 2920 (*s*), 2852 (*m*); ν(C≡N), 1734 (*s*), ν(C—C) + δ(N—H) + δ(C—H), 1635 (*m*), 1440 (*s*), 1380 (*m*); ν(P—C_Ph_), 1097 (*s*); ν(C=S), 1033 (*s*). Ligand IR Data: ν(N—H), 3113 (*m*), ν(C—H), 3078 (*m*); 2981 (*s*); ν(C≡N) 1513 (*s*), δ(N—H), 1467 (*s*), 1381 (*m*); ν(C=S), 1179 (*s*). The compound is partially soluble in di­chloro­methane, but soluble in methanol and chloro­form.

*Preparation of **2***

To the solution of PdCl_2_(PPh_3_)_2_ (0.040 g, 0.060 mmol) in 10 mL of CH_3_CN, was added solid imtH_2_ (0.012 g, 0.120 mmol) followed by the addition of Et_3_N base (0.5 mL). The solution became yellowish orange and was refluxed for 6 h. The orange compound was formed on refluxing and was separated. It was dissolved in a solution of methanol (4 mL) and di­chloro­methane (1 mL) in a culture tube. Slow evaporation of the reaction mixture over a period of one month formed yellowish-orange crystals of compound **2**. Yield: 0.020 g; 69%; m.p. 485–488 K. Analysis found: C, 53.21; H, 3.92; N, 8.48; C_44_H_36_N_6_P_2_Pd_2_S_2_·0.58(CH_3_CN) (1011.5) requires: C, 53.58; H, 3.73; N, 8.36%. IR bands (KBr, cm-1): ν(N—H), 3050 (*m*); ν(C—H), 3081 (*s*), 3005 (*m*), 2968 (*m*), 2938 (*m*); ν(C≡N), 1740 (*s*), d(N—H) + ν(C≡N) + δ(C—H), 1581 (*s*), 1479 (*s*), 1401(s); ν(C=S), 1020 (*m*); ν(P—C_Ph_), 1105 (*s*); Ligand IR data: ν(N—H), 3130 (s), ν(C—H), 2983 (*m*); 2876 (s); ν(C≡N) 1586 (*s*), δ(N—H), 1478 (*s*), 1266 (*m*); ν(C=S), 1120 (*m*). The compound is soluble in methanol, chloro­form and partially in di­chloro­methane.

## 6. Refinement

Crystal data, data collection and structure refinement details are summarized in Table 5 ▸. Hydrogen atoms were fixed geometrically (C—H = 0.93–0.98 Å) with their *U*_iso_(H) = 1.2*U*_eq_(C). The structure of **2** contains partially occupied aceto­nitrile solvent mol­ecules with occupancies of 0.33 and 0.25.

## Supplementary Material

Crystal structure: contains datablock(s) 1, 2. DOI: 10.1107/S2056989023000166/ex2063sup1.cif

Structure factors: contains datablock(s) 1. DOI: 10.1107/S2056989023000166/ex20631sup2.hkl

Structure factors: contains datablock(s) 2. DOI: 10.1107/S2056989023000166/ex20632sup3.hkl

CCDC references: 2234599, 2234598

Additional supporting information:  crystallographic information; 3D view; checkCIF report

## Figures and Tables

**Figure 4:**
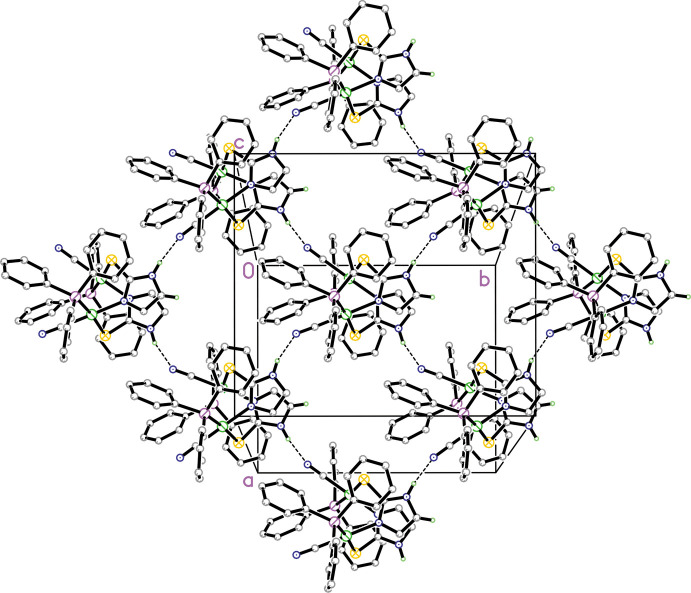
Diagram showing the packing for **2** showing the two inter­molecular 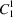 (7) N—H⋯N hydrogen bonding chains in the [110] and [ 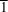 10] directions. Hydrogen atoms not involved in hydrogen bonding are omitted for clarity.

**Table 1:** Selected geometric parameters (Å, °) for **1** [Chem scheme1]

C1—S1	1.728 (2)	N1—Pd1	2.0545 (17)
N3—C8	1.127 (3)	P1—Pd1	2.2861 (6)
C8—Pd1	1.959 (2)	Pd1—S1^i^	2.3547 (6)
			
C8—Pd1—N1	178.31 (8)	C8—Pd1—S1^i^	84.93 (6)
C8—Pd1—P1	87.53 (6)	N1—Pd1—S1^i^	94.24 (5)
N1—Pd1—P1	93.34 (5)	P1—Pd1—S1^i^	172.26 (2)

Symmetry code: (i) 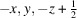 .

**Table 2:** Selected geometric parameters (Å, °) for **2** [Chem scheme1]

Pd1—C1	1.957 (2)	Pd2—P2	2.2984 (5)
Pd1—N11	2.0346 (17)	Pd2—S1	2.3542 (5)
Pd1—P1	2.2914 (5)	S1—C11	1.734 (2)
Pd1—S2	2.3541 (5)	S2—C21	1.733 (2)
Pd2—C2	1.943 (2)	N1—C1	1.143 (3)
Pd2—N21	2.0345 (17)	N2—C2	1.148 (3)
			
C1—Pd1—N11	179.31 (8)	C2—Pd2—N21	178.38 (8)
C1—Pd1—P1	87.19 (6)	C2—Pd2—P2	89.18 (7)
N11—Pd1—P1	92.80 (4)	N21—Pd2—P2	92.44 (5)
C1—Pd1—S2	87.99 (6)	C2—Pd2—S1	86.54 (7)
N11—Pd1—S2	92.06 (5)	N21—Pd2—S1	91.84 (5)
P1—Pd1—S2	173.855 (19)	P2—Pd2—S1	174.489 (19)

**Table 3:** Hydrogen-bond geometry (Å, °) for **1** [Chem scheme1]

*D*—H⋯*A*	*D*—H	H⋯*A*	*D*⋯*A*	*D*—H⋯*A*
N2—H2⋯N3^ii^	0.80 (3)	2.00 (3)	2.796 (3)	177 (3)

Symmetry code: (ii) 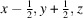 .

**Table 4:** Hydrogen-bond geometry (Å, °) for **2** [Chem scheme1]

*D*—H⋯*A*	*D*—H	H⋯*A*	*D*⋯*A*	*D*—H⋯*A*
N12—H12*A*⋯N2^i^	0.88	1.90	2.770 (3)	169
N22—H22*A*⋯N1^ii^	0.88	1.92	2.760 (3)	160
C12—H13*A*⋯N1*S*	0.95	2.35	3.261 (7)	161
C22—H23*A*⋯N1*T*	0.95	2.29	3.081 (12)	141

Symmetry codes: (i) 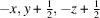 ; (ii) 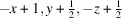 .

**Table 5:** Experimental details

	**1**	**2**
Crystal data		
Chemical formula	[Pd_2_(C_7_H_5_N_2_S)_2_(CN)_2_(C_18_H_15_P)_2_]	[Pd_2_(C_3_H_3_N_2_S)_2_(CN)_2_(C_18_H_15_P)_2_]·0.58C_2_H_3_N
*M*_r_	1087.76	1011.46
Crystal system, space group	Monoclinic, *C*2/*c*	Monoclinic, *P*2_1_/*c*
Temperature (K)	100	110
*a*, *b*, *c* (Å)	13.6026 (12), 13.9719 (12), 25.097 (2)	12.7916 (2), 14.6718 (3), 25.3760 (4)
β (°)	97.417 (1)	101.3491 (15)
*V* (Å^3^)	4729.8 (7)	4669.34 (14)
*Z*	4	4
Radiation type	Mo *K*α	Mo *K*α
μ (mm^−1^)	0.96	0.97
Crystal size (mm)	0.26 × 0.15 × 0.09	0.44 × 0.38 × 0.18

Data collection		
Diffractometer	Bruker SMART APEX CCD	Oxford Diffraction Gemini R (Mo)
Absorption correction	Multi-scan (*SADABS*; Krause *et al.*, 2015 ▸)	Multi-scan (*CrysAlis PRO*; Oxford Diffraction, 2009 ▸)
*T*_min_, *T*_max_	0.811, 0.917	0.962, 1.000
No. of measured, independent and observed [*I* > 2σ(*I*)] reflections	23706, 5857, 5308	15546, 15546, 10002
*R*_int_	0.024	0.031
(sin θ/λ)_max_ (Å^−1^)	0.667	0.761

Refinement		
*R*[*F* ^2^ > 2σ(*F* ^2^)], *wR*(*F* ^2^), *S*	0.028, 0.065, 1.12	0.035, 0.078, 0.95
No. of reflections	5857	15546
No. of parameters	294	561
No. of restraints	0	39
H-atom treatment	H atoms treated by a mixture of independent and constrained refinement	H-atom parameters constrained
Δρ_max_, Δρ_min_ (e Å^−3^)	0.57, −0.89	0.98, −1.20

Computer programs: *APEX2* and *SAINT* (Bruker, 2007 ▸), *CrysAlis RED* and *CrysAlis CCD* (Oxford Diffraction, 2009 ▸), *SHELXS* and *SHELXTL* (Sheldrick, 2008 ▸), *SHELXT* (Sheldrick, 2015*a* ▸), *SHELXL2018/3* (Sheldrick, 2015*b* ▸), and *ShelXle* (Hübschle *et al.*, 2011 ▸).

## supplementary crystallographic information

### Bis(µ-1H-benzimidazole-2-thiolato)-κ2N3:S;κ2S:N3-bis[cyanido(triphenylphosphine-κP)palladium(II)] (1) Crystal data

**Table:**

[Pd_2_(C_7_H_5_N_2_S)_2_(CN)_2_(C_18_H_15_P)_2_]	*F*(000) = 2192
*M**_r_* = 1087.76	*D*_x_ = 1.528 Mg m^−^^3^
Monoclinic, *C*2/*c*	Mo *K*α radiation, λ = 0.71073 Å
*a* = 13.6026 (12) Å	Cell parameters from 9977 reflections
*b* = 13.9719 (12) Å	θ = 2.2–31.8°
*c* = 25.097 (2) Å	µ = 0.96 mm^−^^1^
β = 97.417 (1)°	*T* = 100 K
*V* = 4729.8 (7) Å^3^	Block, orange
*Z* = 4	0.26 × 0.15 × 0.09 mm

### Bis(µ-1H-benzimidazole-2-thiolato)-κ2N3:S;κ2S:N3-bis[cyanido(triphenylphosphine-κP)palladium(II)] (1) Data collection

**Table:**

Bruker SMART APEX CCD diffractometer	5857 independent reflections
Radiation source: fine-focus sealed tube	5308 reflections with *I* > 2σ(*I*)
Graphite monochromator	*R*_int_ = 0.024
ω scans	θ_max_ = 28.3°, θ_min_ = 1.6°
Absorption correction: multi-scan (SADABS; Krause *et al.*, 2015)	*h* = −18→18
*T*_min_ = 0.811, *T*_max_ = 0.917	*k* = −18→18
23706 measured reflections	*l* = −33→33

### Bis(µ-1H-benzimidazole-2-thiolato)-κ2N3:S;κ2S:N3-bis[cyanido(triphenylphosphine-κP)palladium(II)] (1) Refinement

**Table:**

Refinement on *F*^2^	Primary atom site location: structure-invariant direct methods
Least-squares matrix: full	Secondary atom site location: difference Fourier map
*R*[*F*^2^ > 2σ(*F*^2^)] = 0.028	Hydrogen site location: mixed
*wR*(*F*^2^) = 0.065	H atoms treated by a mixture of independent and constrained refinement
*S* = 1.12	*w* = 1/[σ^2^(*F*_o_^2^) + (0.0241*P*)^2^ + 9.9394*P*] where *P* = (*F*_o_^2^ + 2*F*_c_^2^)/3
5857 reflections	(Δ/σ)_max_ < 0.001
294 parameters	Δρ_max_ = 0.57 e Å^−^^3^
0 restraints	Δρ_min_ = −0.89 e Å^−^^3^

### Bis(µ-1H-benzimidazole-2-thiolato)-κ2N3:S;κ2S:N3-bis[cyanido(triphenylphosphine-κP)palladium(II)] (1) Special details

**Table:**

Geometry. All esds (except the esd in the dihedral angle between two l.s. planes) are estimated using the full covariance matrix. The cell esds are taken into account individually in the estimation of esds in distances, angles and torsion angles; correlations between esds in cell parameters are only used when they are defined by crystal symmetry. An approximate (isotropic) treatment of cell esds is used for estimating esds involving l.s. planes.

### Bis(µ-1H-benzimidazole-2-thiolato)-κ2N3:S;κ2S:N3-bis[cyanido(triphenylphosphine-κP)palladium(II)] (1) Fractional atomic coordinates and isotropic or equivalent isotropic displacement parameters (Å^2^)

**Table:**

	*x*	*y*	*z*	*U*_iso_*/*U*_eq_	
C1	−0.07279 (15)	0.26650 (15)	0.30035 (8)	0.0167 (4)	
C2	−0.02067 (16)	0.41441 (16)	0.31826 (8)	0.0204 (4)	
N3	0.20989 (15)	−0.05673 (15)	0.29010 (8)	0.0296 (4)	
C3	−0.00951 (19)	0.51282 (17)	0.32406 (10)	0.0285 (5)	
H3	−0.065219	0.554027	0.323359	0.034*	
C4	0.0864 (2)	0.54824 (18)	0.33093 (10)	0.0333 (6)	
H4	0.096907	0.615208	0.334994	0.040*	
C5	0.16821 (19)	0.48698 (18)	0.33198 (9)	0.0290 (5)	
H5	0.233000	0.513645	0.336220	0.035*	
C6	0.15735 (17)	0.38872 (16)	0.32701 (8)	0.0223 (4)	
H6	0.213217	0.347564	0.328414	0.027*	
C7	0.06114 (16)	0.35283 (15)	0.31987 (8)	0.0184 (4)	
C8	0.17115 (16)	0.01314 (17)	0.29575 (9)	0.0224 (4)	
C9	−0.02560 (16)	0.14880 (15)	0.41389 (8)	0.0192 (4)	
C10	−0.12021 (17)	0.11322 (17)	0.41677 (9)	0.0245 (5)	
H10	−0.135247	0.048473	0.407466	0.029*	
C11	−0.19289 (18)	0.1723 (2)	0.43325 (10)	0.0319 (5)	
H11	−0.257352	0.147566	0.435347	0.038*	
C12	−0.1718 (2)	0.26638 (19)	0.44654 (10)	0.0323 (6)	
H12	−0.222246	0.307008	0.456694	0.039*	
C13	−0.0770 (2)	0.30173 (18)	0.44508 (10)	0.0303 (5)	
H13	−0.061902	0.366039	0.455393	0.036*	
C14	−0.00422 (18)	0.24371 (17)	0.42866 (9)	0.0244 (5)	
H14	0.060499	0.268435	0.427421	0.029*	
C15	0.17398 (16)	0.07885 (16)	0.43693 (9)	0.0221 (4)	
C16	0.26896 (17)	0.06387 (18)	0.42373 (10)	0.0289 (5)	
H16	0.279017	0.056685	0.387184	0.035*	
C17	0.34938 (19)	0.05938 (19)	0.46410 (12)	0.0361 (6)	
H17	0.414124	0.048636	0.454978	0.043*	
C18	0.3355 (2)	0.07046 (19)	0.51719 (11)	0.0373 (6)	
H18	0.390378	0.065693	0.544619	0.045*	
C19	0.2424 (2)	0.0884 (2)	0.53042 (10)	0.0374 (6)	
H19	0.233304	0.098140	0.566924	0.045*	
C20	0.16164 (19)	0.0923 (2)	0.49069 (9)	0.0318 (5)	
H20	0.097369	0.104236	0.500179	0.038*	
C21	0.02075 (16)	−0.04251 (15)	0.38040 (9)	0.0205 (4)	
C22	0.04218 (19)	−0.10869 (17)	0.42181 (9)	0.0274 (5)	
H22	0.083356	−0.091135	0.453803	0.033*	
C23	0.0030 (2)	−0.20054 (18)	0.41604 (11)	0.0367 (6)	
H23	0.017564	−0.245817	0.444194	0.044*	
C24	−0.0571 (2)	−0.22642 (18)	0.36955 (11)	0.0351 (6)	
H24	−0.084438	−0.289039	0.366194	0.042*	
C25	−0.07770 (18)	−0.16150 (18)	0.32787 (10)	0.0293 (5)	
H25	−0.118226	−0.179735	0.295793	0.035*	
C26	−0.03879 (16)	−0.06974 (16)	0.33325 (9)	0.0239 (5)	
H26	−0.052690	−0.025117	0.304710	0.029*	
N1	0.02621 (12)	0.26021 (12)	0.30959 (7)	0.0160 (3)	
N2	−0.10313 (14)	0.35712 (13)	0.30663 (7)	0.0200 (4)	
H2	−0.157 (2)	0.3796 (18)	0.3021 (11)	0.024*	
P1	0.06743 (4)	0.07947 (4)	0.38495 (2)	0.01712 (11)	
Pd1	0.10174 (2)	0.13416 (2)	0.30350 (2)	0.01536 (5)	
S1	−0.15249 (4)	0.17333 (4)	0.28003 (2)	0.01886 (11)	

### Bis(µ-1H-benzimidazole-2-thiolato)-κ2N3:S;κ2S:N3-bis[cyanido(triphenylphosphine-κP)palladium(II)] (1) Atomic displacement parameters (Å^2^)

**Table:**

	*U*^11^	*U*^22^	*U*^33^	*U*^12^	*U*^13^	*U*^23^
C1	0.0176 (10)	0.0189 (10)	0.0133 (9)	0.0014 (8)	0.0016 (7)	−0.0006 (7)
C2	0.0239 (11)	0.0209 (11)	0.0158 (10)	−0.0007 (8)	0.0009 (8)	−0.0011 (8)
N3	0.0284 (10)	0.0308 (11)	0.0294 (11)	0.0092 (9)	0.0022 (8)	0.0032 (9)
C3	0.0346 (13)	0.0214 (11)	0.0289 (12)	0.0029 (10)	0.0026 (10)	−0.0027 (9)
C4	0.0457 (15)	0.0225 (12)	0.0308 (13)	−0.0079 (11)	0.0011 (11)	−0.0064 (10)
C5	0.0305 (13)	0.0318 (13)	0.0236 (11)	−0.0118 (10)	−0.0005 (9)	−0.0013 (10)
C6	0.0215 (10)	0.0272 (11)	0.0175 (10)	−0.0029 (9)	−0.0006 (8)	−0.0007 (8)
C7	0.0213 (10)	0.0207 (10)	0.0128 (9)	−0.0013 (8)	0.0011 (7)	−0.0001 (7)
C8	0.0187 (10)	0.0307 (12)	0.0179 (10)	0.0036 (9)	0.0026 (8)	0.0011 (9)
C9	0.0214 (10)	0.0225 (11)	0.0140 (9)	0.0036 (8)	0.0032 (8)	0.0004 (8)
C10	0.0246 (11)	0.0285 (12)	0.0208 (10)	−0.0005 (9)	0.0053 (8)	−0.0025 (9)
C11	0.0218 (11)	0.0448 (15)	0.0302 (13)	0.0023 (11)	0.0076 (9)	−0.0033 (11)
C12	0.0343 (14)	0.0378 (14)	0.0264 (12)	0.0136 (11)	0.0103 (10)	−0.0011 (10)
C13	0.0422 (14)	0.0262 (12)	0.0246 (12)	0.0047 (11)	0.0117 (10)	−0.0031 (9)
C14	0.0277 (12)	0.0257 (11)	0.0208 (11)	−0.0007 (9)	0.0074 (9)	−0.0006 (9)
C15	0.0217 (10)	0.0221 (11)	0.0213 (11)	−0.0006 (8)	−0.0016 (8)	0.0029 (8)
C16	0.0231 (11)	0.0321 (13)	0.0304 (12)	0.0034 (10)	−0.0012 (9)	−0.0043 (10)
C17	0.0240 (12)	0.0332 (14)	0.0480 (16)	0.0037 (10)	−0.0075 (11)	−0.0064 (12)
C18	0.0366 (14)	0.0310 (13)	0.0385 (15)	−0.0026 (11)	−0.0174 (11)	0.0020 (11)
C19	0.0425 (15)	0.0458 (16)	0.0214 (12)	−0.0073 (13)	−0.0058 (11)	0.0062 (11)
C20	0.0277 (12)	0.0449 (15)	0.0217 (11)	−0.0019 (11)	−0.0003 (9)	0.0048 (11)
C21	0.0207 (10)	0.0186 (10)	0.0231 (11)	0.0032 (8)	0.0062 (8)	0.0013 (8)
C22	0.0370 (13)	0.0237 (11)	0.0220 (11)	0.0045 (10)	0.0062 (10)	0.0021 (9)
C23	0.0587 (18)	0.0229 (12)	0.0297 (13)	0.0036 (12)	0.0106 (12)	0.0055 (10)
C24	0.0473 (16)	0.0206 (12)	0.0399 (15)	−0.0035 (11)	0.0154 (12)	−0.0047 (10)
C25	0.0275 (12)	0.0268 (12)	0.0338 (13)	−0.0018 (10)	0.0049 (10)	−0.0062 (10)
C26	0.0230 (11)	0.0229 (11)	0.0252 (11)	0.0015 (9)	0.0009 (9)	0.0001 (9)
N1	0.0154 (8)	0.0176 (8)	0.0150 (8)	−0.0001 (6)	0.0018 (6)	−0.0001 (6)
N2	0.0181 (9)	0.0213 (9)	0.0204 (9)	0.0049 (7)	0.0020 (7)	−0.0011 (7)
P1	0.0167 (2)	0.0192 (3)	0.0156 (2)	0.0018 (2)	0.00211 (19)	0.00103 (19)
Pd1	0.01371 (8)	0.01746 (8)	0.01506 (8)	0.00304 (6)	0.00240 (5)	0.00079 (6)
S1	0.0167 (2)	0.0223 (3)	0.0181 (2)	−0.00317 (19)	0.00424 (18)	−0.00229 (19)

### Bis(µ-1H-benzimidazole-2-thiolato)-κ2N3:S;κ2S:N3-bis[cyanido(triphenylphosphine-κP)palladium(II)] (1) Geometric parameters (Å, º)

**Table:**

C1—N1	1.339 (3)	C15—C16	1.390 (3)
C1—N2	1.347 (3)	C15—C20	1.394 (3)
C1—S1	1.728 (2)	C15—P1	1.820 (2)
C2—N2	1.378 (3)	C16—C17	1.393 (3)
C2—C3	1.389 (3)	C16—H16	0.9500
C2—C7	1.403 (3)	C17—C18	1.378 (4)
N3—C8	1.127 (3)	C17—H17	0.9500
C3—C4	1.385 (4)	C18—C19	1.374 (4)
C3—H3	0.9500	C18—H18	0.9500
C4—C5	1.401 (4)	C19—C20	1.386 (3)
C4—H4	0.9500	C19—H19	0.9500
C5—C6	1.385 (3)	C20—H20	0.9500
C5—H5	0.9500	C21—C22	1.394 (3)
C6—C7	1.391 (3)	C21—C26	1.398 (3)
C6—H6	0.9500	C21—P1	1.817 (2)
C7—N1	1.391 (3)	C22—C23	1.390 (4)
C8—Pd1	1.959 (2)	C22—H22	0.9500
C9—C10	1.390 (3)	C23—C24	1.384 (4)
C9—C14	1.397 (3)	C23—H23	0.9500
C9—P1	1.818 (2)	C24—C25	1.386 (4)
C10—C11	1.391 (3)	C24—H24	0.9500
C10—H10	0.9500	C25—C26	1.387 (3)
C11—C12	1.378 (4)	C25—H25	0.9500
C11—H11	0.9500	C26—H26	0.9500
C12—C13	1.387 (4)	N1—Pd1	2.0545 (17)
C12—H12	0.9500	N2—H2	0.80 (3)
C13—C14	1.382 (3)	P1—Pd1	2.2861 (6)
C13—H13	0.9500	Pd1—S1^i^	2.3547 (6)
C14—H14	0.9500		
			
N1—C1—N2	110.96 (18)	C16—C17—H17	119.8
N1—C1—S1	125.44 (16)	C19—C18—C17	120.0 (2)
N2—C1—S1	123.53 (16)	C19—C18—H18	120.0
N2—C2—C3	132.2 (2)	C17—C18—H18	120.0
N2—C2—C7	105.70 (19)	C18—C19—C20	120.2 (3)
C3—C2—C7	121.9 (2)	C18—C19—H19	119.9
C4—C3—C2	117.1 (2)	C20—C19—H19	119.9
C4—C3—H3	121.5	C19—C20—C15	120.5 (2)
C2—C3—H3	121.5	C19—C20—H20	119.7
C3—C4—C5	121.1 (2)	C15—C20—H20	119.7
C3—C4—H4	119.4	C22—C21—C26	119.6 (2)
C5—C4—H4	119.4	C22—C21—P1	122.52 (18)
C6—C5—C4	121.9 (2)	C26—C21—P1	117.92 (16)
C6—C5—H5	119.0	C23—C22—C21	119.6 (2)
C4—C5—H5	119.0	C23—C22—H22	120.2
C5—C6—C7	117.2 (2)	C21—C22—H22	120.2
C5—C6—H6	121.4	C24—C23—C22	120.4 (2)
C7—C6—H6	121.4	C24—C23—H23	119.8
N1—C7—C6	130.7 (2)	C22—C23—H23	119.8
N1—C7—C2	108.33 (18)	C23—C24—C25	120.4 (2)
C6—C7—C2	120.8 (2)	C23—C24—H24	119.8
N3—C8—Pd1	178.4 (2)	C25—C24—H24	119.8
C10—C9—C14	119.2 (2)	C24—C25—C26	119.6 (2)
C10—C9—P1	121.81 (17)	C24—C25—H25	120.2
C14—C9—P1	118.68 (17)	C26—C25—H25	120.2
C9—C10—C11	120.1 (2)	C25—C26—C21	120.4 (2)
C9—C10—H10	119.9	C25—C26—H26	119.8
C11—C10—H10	119.9	C21—C26—H26	119.8
C12—C11—C10	120.3 (2)	C1—N1—C7	106.42 (17)
C12—C11—H11	119.8	C1—N1—Pd1	123.00 (14)
C10—C11—H11	119.8	C7—N1—Pd1	130.47 (14)
C11—C12—C13	119.9 (2)	C1—N2—C2	108.50 (18)
C11—C12—H12	120.0	C1—N2—H2	130.2 (19)
C13—C12—H12	120.0	C2—N2—H2	121.1 (19)
C14—C13—C12	120.2 (2)	C21—P1—C9	105.62 (10)
C14—C13—H13	119.9	C21—P1—C15	106.27 (10)
C12—C13—H13	119.9	C9—P1—C15	104.31 (10)
C13—C14—C9	120.2 (2)	C21—P1—Pd1	111.54 (7)
C13—C14—H14	119.9	C9—P1—Pd1	114.34 (7)
C9—C14—H14	119.9	C15—P1—Pd1	114.00 (7)
C16—C15—C20	118.9 (2)	C8—Pd1—N1	178.31 (8)
C16—C15—P1	120.56 (17)	C8—Pd1—P1	87.53 (6)
C20—C15—P1	120.58 (18)	N1—Pd1—P1	93.34 (5)
C15—C16—C17	120.0 (2)	C8—Pd1—S1^i^	84.93 (6)
C15—C16—H16	120.0	N1—Pd1—S1^i^	94.24 (5)
C17—C16—H16	120.0	P1—Pd1—S1^i^	172.26 (2)
C18—C17—C16	120.4 (3)	C1—S1—Pd1^i^	101.12 (7)
C18—C17—H17	119.8		
			
N2—C2—C3—C4	−174.0 (2)	P1—C21—C26—C25	−179.03 (18)
C7—C2—C3—C4	0.8 (3)	N2—C1—N1—C7	−2.9 (2)
C2—C3—C4—C5	−0.1 (4)	S1—C1—N1—C7	174.31 (15)
C3—C4—C5—C6	−0.9 (4)	N2—C1—N1—Pd1	−179.43 (13)
C4—C5—C6—C7	1.2 (3)	S1—C1—N1—Pd1	−2.2 (2)
C5—C6—C7—N1	174.3 (2)	C6—C7—N1—C1	−173.3 (2)
C5—C6—C7—C2	−0.5 (3)	C2—C7—N1—C1	2.0 (2)
N2—C2—C7—N1	−0.4 (2)	C6—C7—N1—Pd1	2.9 (3)
C3—C2—C7—N1	−176.4 (2)	C2—C7—N1—Pd1	178.18 (14)
N2—C2—C7—C6	175.44 (19)	N1—C1—N2—C2	2.7 (2)
C3—C2—C7—C6	−0.5 (3)	S1—C1—N2—C2	−174.57 (15)
C14—C9—C10—C11	1.2 (3)	C3—C2—N2—C1	174.0 (2)
P1—C9—C10—C11	−172.37 (18)	C7—C2—N2—C1	−1.3 (2)
C9—C10—C11—C12	0.3 (4)	C22—C21—P1—C9	−90.0 (2)
C10—C11—C12—C13	−2.0 (4)	C26—C21—P1—C9	90.01 (18)
C11—C12—C13—C14	2.1 (4)	C22—C21—P1—C15	20.4 (2)
C12—C13—C14—C9	−0.6 (4)	C26—C21—P1—C15	−159.57 (17)
C10—C9—C14—C13	−1.0 (3)	C22—C21—P1—Pd1	145.20 (17)
P1—C9—C14—C13	172.69 (18)	C26—C21—P1—Pd1	−34.77 (19)
C20—C15—C16—C17	2.1 (4)	C10—C9—P1—C21	−14.5 (2)
P1—C15—C16—C17	−177.2 (2)	C14—C9—P1—C21	171.94 (17)
C15—C16—C17—C18	−0.5 (4)	C10—C9—P1—C15	−126.31 (19)
C16—C17—C18—C19	−1.6 (4)	C14—C9—P1—C15	60.13 (19)
C17—C18—C19—C20	2.1 (4)	C10—C9—P1—Pd1	108.52 (18)
C18—C19—C20—C15	−0.5 (4)	C14—C9—P1—Pd1	−65.04 (18)
C16—C15—C20—C19	−1.6 (4)	C16—C15—P1—C21	92.0 (2)
P1—C15—C20—C19	177.7 (2)	C20—C15—P1—C21	−87.2 (2)
C26—C21—C22—C23	−0.9 (3)	C16—C15—P1—C9	−156.63 (19)
P1—C21—C22—C23	179.13 (19)	C20—C15—P1—C9	24.1 (2)
C21—C22—C23—C24	−0.1 (4)	C16—C15—P1—Pd1	−31.2 (2)
C22—C23—C24—C25	1.0 (4)	C20—C15—P1—Pd1	149.50 (18)
C23—C24—C25—C26	−0.9 (4)	N1—C1—S1—Pd1^i^	−61.89 (18)
C24—C25—C26—C21	−0.1 (4)	N2—C1—S1—Pd1^i^	114.98 (17)
C22—C21—C26—C25	1.0 (3)		

Symmetry code: (i) −*x*, *y*, −*z*+1/2.

### Bis(µ-1H-benzimidazole-2-thiolato)-κ2N3:S;κ2S:N3-bis[cyanido(triphenylphosphine-κP)palladium(II)] (1) Hydrogen-bond geometry (Å, º)

**Table:**

*D*—H···*A*	*D*—H	H···*A*	*D*···*A*	*D*—H···*A*
N2—H2···N3^ii^	0.80 (3)	2.00 (3)	2.796 (3)	177 (3)

Symmetry code: (ii) *x*−1/2, *y*+1/2, *z*.

### \ Bis(µ-1H-imidazole-2-thiolato)-κ2N3:S;\ κ2S:N3-bis[cyanido(triphenylphosphine-κP)\ palladium(II)] acetonitrile 0.58-solvate (2) Crystal data

**Table:**

[Pd_2_(C_3_H_3_N_2_S)_2_(CN)_2_(C_18_H_15_P)_2_]·0.58C_2_H_3_N	*F*(000) = 2035
*M**_r_* = 1011.46	*D*_x_ = 1.439 Mg m^−^^3^
Monoclinic, *P*2_1_/*c*	Mo *K*α radiation, λ = 0.71073 Å
*a* = 12.7916 (2) Å	Cell parameters from 15979 reflections
*b* = 14.6718 (3) Å	θ = 4.7–32.7°
*c* = 25.3760 (4) Å	µ = 0.97 mm^−^^1^
β = 101.3491 (15)°	*T* = 110 K
*V* = 4669.34 (14) Å^3^	Plate, yellow
*Z* = 4	0.44 × 0.38 × 0.18 mm

### \ Bis(µ-1H-imidazole-2-thiolato)-κ2N3:S;\ κ2S:N3-bis[cyanido(triphenylphosphine-κP)\ palladium(II)] acetonitrile 0.58-solvate (2) Data collection

**Table:**

Oxford Diffraction Gemini R (Mo) diffractometer	15546 independent reflections
Graphite monochromator	10002 reflections with *I* > 2σ(*I*)
Detector resolution: 10.5081 pixels mm^-1^	*R*_int_ = 0.031
ω scans	θ_max_ = 32.8°, θ_min_ = 4.7°
Absorption correction: multi-scan (CrysAlisPro; Oxford Diffraction, 2009)	*h* = −18→18
*T*_min_ = 0.962, *T*_max_ = 1.000	*k* = −17→22
15546 measured reflections	*l* = −34→36

### \ Bis(µ-1H-imidazole-2-thiolato)-κ2N3:S;\ κ2S:N3-bis[cyanido(triphenylphosphine-κP)\ palladium(II)] acetonitrile 0.58-solvate (2) Refinement

**Table:**

Refinement on *F*^2^	Primary atom site location: structure-invariant direct methods
Least-squares matrix: full	Secondary atom site location: difference Fourier map
*R*[*F*^2^ > 2σ(*F*^2^)] = 0.035	Hydrogen site location: inferred from neighbouring sites
*wR*(*F*^2^) = 0.078	H-atom parameters constrained
*S* = 0.95	*w* = 1/[σ^2^(*F*_o_^2^) + (0.0359*P*)^2^] where *P* = (*F*_o_^2^ + 2*F*_c_^2^)/3
15546 reflections	(Δ/σ)_max_ = 0.002
561 parameters	Δρ_max_ = 0.98 e Å^−^^3^
39 restraints	Δρ_min_ = −1.20 e Å^−^^3^

### \ Bis(µ-1H-imidazole-2-thiolato)-κ2N3:S;\ κ2S:N3-bis[cyanido(triphenylphosphine-κP)\ palladium(II)] acetonitrile 0.58-solvate (2) Special details

**Table:**

Geometry. All esds (except the esd in the dihedral angle between two l.s. planes) are estimated using the full covariance matrix. The cell esds are taken into account individually in the estimation of esds in distances, angles and torsion angles; correlations between esds in cell parameters are only used when they are defined by crystal symmetry. An approximate (isotropic) treatment of cell esds is used for estimating esds involving l.s. planes.

### \ Bis(µ-1H-imidazole-2-thiolato)-κ2N3:S;\ κ2S:N3-bis[cyanido(triphenylphosphine-κP)\ palladium(II)] acetonitrile 0.58-solvate (2) Fractional atomic coordinates and isotropic or equivalent isotropic displacement parameters (Å^2^)

**Table:**

	*x*	*y*	*z*	*U*_iso_*/*U*_eq_	Occ. (<1)
Pd1	0.35095 (2)	0.87099 (2)	0.30663 (2)	0.02356 (4)	
Pd2	0.14320 (2)	0.88485 (2)	0.19323 (2)	0.02559 (4)	
S1	0.08912 (4)	0.94151 (4)	0.27044 (2)	0.03170 (12)	
S2	0.41813 (4)	0.90816 (4)	0.22938 (2)	0.03362 (12)	
P1	0.30170 (4)	0.82559 (4)	0.38464 (2)	0.02500 (11)	
P2	0.17961 (4)	0.82426 (4)	0.11528 (2)	0.03002 (12)	
N1	0.43502 (18)	0.67452 (15)	0.29819 (7)	0.0548 (6)	
N2	0.0059 (2)	0.71602 (18)	0.20621 (8)	0.0727 (8)	
N11	0.29215 (13)	0.99892 (11)	0.31143 (6)	0.0272 (4)	
N12	0.17481 (16)	1.10817 (13)	0.30467 (7)	0.0410 (5)	
H12A	0.113398	1.137168	0.298069	0.049*	
N21	0.23236 (13)	0.99792 (12)	0.18750 (6)	0.0284 (4)	
N22	0.37399 (16)	1.08292 (14)	0.19348 (6)	0.0416 (5)	
H22A	0.441192	1.100160	0.199723	0.050*	
C1	0.40581 (17)	0.74749 (16)	0.30153 (7)	0.0363 (5)	
C2	0.05709 (19)	0.77821 (17)	0.20056 (7)	0.0412 (6)	
C11	0.18880 (16)	1.01847 (15)	0.29650 (7)	0.0291 (5)	
C12	0.2718 (2)	1.14654 (17)	0.32489 (8)	0.0480 (7)	
H13A	0.285290	1.208633	0.334479	0.058*	
C13	0.3447 (2)	1.07915 (16)	0.32859 (8)	0.0385 (5)	
H14A	0.419358	1.085725	0.340892	0.046*	
C21	0.33859 (16)	0.99883 (15)	0.20226 (7)	0.0311 (5)	
C22	0.2886 (2)	1.13701 (17)	0.17333 (9)	0.0484 (7)	
H23A	0.290465	1.199438	0.163621	0.058*	
C23	0.20113 (19)	1.08439 (16)	0.16993 (8)	0.0381 (5)	
H24A	0.129679	1.103756	0.157463	0.046*	
C1A	0.24085 (17)	0.71348 (15)	0.37962 (7)	0.0324 (5)	
C2A	0.16469 (19)	0.69425 (17)	0.33331 (8)	0.0424 (6)	
H2AA	0.148149	0.738927	0.305829	0.051*	
C3A	0.1134 (2)	0.61103 (19)	0.32715 (9)	0.0575 (8)	
H3AA	0.060788	0.599083	0.295887	0.069*	
C4A	0.1386 (3)	0.54504 (19)	0.36657 (10)	0.0654 (9)	
H4AA	0.102770	0.487976	0.362623	0.079*	
C5A	0.2166 (2)	0.56257 (18)	0.41211 (9)	0.0563 (8)	
H5AA	0.235385	0.516640	0.438678	0.068*	
C6A	0.26698 (19)	0.64645 (16)	0.41884 (8)	0.0383 (5)	
H6AA	0.319336	0.658288	0.450210	0.046*	
C1B	0.41603 (16)	0.82116 (14)	0.44038 (7)	0.0295 (4)	
C2B	0.51745 (18)	0.80590 (18)	0.43012 (8)	0.0442 (6)	
H2BA	0.527274	0.798236	0.394240	0.053*	
C3B	0.6042 (2)	0.8019 (2)	0.47257 (9)	0.0557 (8)	
H3BA	0.673349	0.790636	0.465621	0.067*	
C4B	0.5911 (2)	0.81419 (18)	0.52455 (9)	0.0457 (6)	
H4BA	0.651230	0.811916	0.553268	0.055*	
C5B	0.4920 (2)	0.82962 (18)	0.53501 (8)	0.0454 (6)	
H5BA	0.483094	0.838279	0.570956	0.054*	
C6B	0.40439 (18)	0.83263 (17)	0.49303 (7)	0.0398 (6)	
H6BA	0.335423	0.842714	0.500531	0.048*	
C1C	0.20732 (16)	0.89953 (15)	0.40931 (7)	0.0286 (4)	
C2C	0.23993 (18)	0.98632 (16)	0.42808 (8)	0.0363 (5)	
H2CA	0.311953	1.004513	0.430333	0.044*	
C3C	0.1672 (2)	1.04648 (18)	0.44355 (8)	0.0457 (6)	
H3CA	0.189705	1.105585	0.456258	0.055*	
C4C	0.0636 (2)	1.0207 (2)	0.44049 (9)	0.0533 (7)	
H4CA	0.013952	1.062039	0.450704	0.064*	
C5C	0.0311 (2)	0.9349 (2)	0.42266 (11)	0.0607 (8)	
H5CA	−0.040582	0.916511	0.421319	0.073*	
C6C	0.10260 (19)	0.87539 (18)	0.40664 (9)	0.0446 (6)	
H6CA	0.078935	0.816741	0.393571	0.054*	
C1D	0.28204 (18)	0.88581 (15)	0.08915 (7)	0.0335 (5)	
C2D	0.25910 (19)	0.97236 (17)	0.06660 (8)	0.0403 (6)	
H2DA	0.188431	0.995509	0.061180	0.048*	
C3D	0.3395 (2)	1.02420 (19)	0.05223 (8)	0.0494 (7)	
H3DA	0.323656	1.083075	0.037180	0.059*	
C4D	0.4415 (2)	0.9915 (2)	0.05946 (9)	0.0533 (7)	
H4DA	0.496407	1.027784	0.049892	0.064*	
C5D	0.4644 (2)	0.9055 (2)	0.08075 (11)	0.0566 (7)	
H5DA	0.534715	0.881930	0.084976	0.068*	
C6D	0.3844 (2)	0.85306 (18)	0.09605 (9)	0.0453 (6)	
H6DA	0.400780	0.794418	0.111328	0.054*	
C1E	0.06406 (17)	0.82453 (15)	0.05978 (7)	0.0334 (5)	
C2E	0.07759 (19)	0.82814 (18)	0.00681 (8)	0.0446 (6)	
H2EA	0.147239	0.832430	−0.000792	0.054*	
C3E	−0.0108 (2)	0.82547 (19)	−0.03519 (8)	0.0497 (7)	
H3EA	−0.001292	0.828234	−0.071351	0.060*	
C4E	−0.1110 (2)	0.81890 (18)	−0.02460 (8)	0.0459 (6)	
H4EA	−0.170923	0.816583	−0.053380	0.055*	
C5E	−0.12546 (19)	0.81559 (18)	0.02794 (8)	0.0446 (6)	
H5EA	−0.195388	0.811418	0.035189	0.054*	
C6E	−0.03805 (18)	0.81836 (16)	0.07012 (8)	0.0375 (5)	
H6EA	−0.048286	0.816009	0.106170	0.045*	
C1F	0.22604 (18)	0.70707 (15)	0.12111 (7)	0.0351 (5)	
C2F	0.20602 (18)	0.64505 (16)	0.07863 (8)	0.0379 (5)	
H2FA	0.162463	0.662691	0.045431	0.045*	
C3F	0.2487 (2)	0.55839 (18)	0.08427 (9)	0.0477 (6)	
H3FA	0.233104	0.516470	0.055249	0.057*	
C4F	0.3142 (2)	0.53223 (18)	0.13203 (10)	0.0546 (7)	
H4FA	0.345070	0.473104	0.135597	0.066*	
C5F	0.3342 (2)	0.59283 (19)	0.17436 (10)	0.0614 (8)	
H5FA	0.378526	0.575078	0.207321	0.074*	
C6F	0.2903 (2)	0.67900 (18)	0.16923 (9)	0.0513 (7)	
H6FA	0.303984	0.719733	0.198891	0.062*	
N1S	0.2657 (6)	1.3556 (5)	0.3677 (3)	0.0700 (15)	0.33
C1S	0.2748 (8)	1.4030 (6)	0.3321 (4)	0.0744 (14)	0.33
C2S	0.2930 (8)	1.4590 (7)	0.2900 (4)	0.0818 (15)	0.33
H2S1	0.334222	1.512578	0.304829	0.123*	0.33
H2S2	0.224550	1.478602	0.268390	0.123*	0.33
H2S3	0.332939	1.424861	0.267223	0.123*	0.33
N1T	0.1809 (10)	1.3230 (8)	0.1440 (5)	0.0995 (19)	0.25
C1T	0.1767 (12)	1.3567 (10)	0.1851 (5)	0.0967 (18)	0.25
C2T	0.1924 (12)	1.4067 (10)	0.2333 (5)	0.0912 (17)	0.25
H2T1	0.268941	1.410818	0.248353	0.137*	0.25
H2T2	0.156359	1.375999	0.259002	0.137*	0.25
H2T3	0.163014	1.468107	0.226043	0.137*	0.25

### \ Bis(µ-1H-imidazole-2-thiolato)-κ2N3:S;\ κ2S:N3-bis[cyanido(triphenylphosphine-κP)\ palladium(II)] acetonitrile 0.58-solvate (2) Atomic displacement parameters (Å^2^)

**Table:**

	*U*^11^	*U*^22^	*U*^33^	*U*^12^	*U*^13^	*U*^23^
Pd1	0.02286 (7)	0.03057 (9)	0.01702 (7)	0.00603 (6)	0.00335 (5)	0.00011 (6)
Pd2	0.02643 (8)	0.03380 (9)	0.01603 (7)	−0.00787 (7)	0.00293 (5)	0.00017 (6)
S1	0.0255 (3)	0.0476 (4)	0.0223 (2)	0.0063 (2)	0.00533 (18)	0.0017 (2)
S2	0.0291 (3)	0.0485 (4)	0.0253 (2)	−0.0039 (3)	0.01024 (19)	−0.0011 (2)
P1	0.0293 (3)	0.0286 (3)	0.0164 (2)	0.0046 (2)	0.00299 (18)	0.00043 (19)
P2	0.0365 (3)	0.0359 (3)	0.0171 (2)	−0.0075 (3)	0.00384 (19)	−0.0021 (2)
N1	0.0692 (15)	0.0542 (14)	0.0369 (11)	0.0373 (12)	0.0003 (9)	−0.0038 (10)
N2	0.0887 (18)	0.0875 (19)	0.0348 (11)	−0.0607 (16)	−0.0051 (11)	0.0123 (11)
N11	0.0345 (9)	0.0289 (9)	0.0172 (7)	0.0039 (8)	0.0028 (6)	−0.0003 (6)
N12	0.0564 (13)	0.0378 (11)	0.0256 (9)	0.0245 (10)	0.0002 (8)	−0.0022 (8)
N21	0.0337 (9)	0.0324 (10)	0.0183 (7)	−0.0077 (8)	0.0034 (6)	0.0014 (7)
N22	0.0480 (12)	0.0498 (13)	0.0247 (9)	−0.0249 (10)	0.0017 (8)	0.0027 (8)
C1	0.0385 (12)	0.0475 (14)	0.0213 (10)	0.0216 (11)	0.0018 (8)	−0.0009 (9)
C2	0.0484 (14)	0.0545 (16)	0.0182 (9)	−0.0221 (12)	0.0001 (9)	0.0018 (9)
C11	0.0373 (11)	0.0330 (12)	0.0166 (8)	0.0129 (10)	0.0039 (7)	0.0013 (8)
C12	0.0769 (19)	0.0327 (13)	0.0291 (11)	0.0036 (13)	−0.0023 (11)	−0.0074 (10)
C13	0.0486 (14)	0.0383 (13)	0.0247 (10)	−0.0040 (11)	−0.0020 (9)	−0.0030 (9)
C21	0.0383 (12)	0.0382 (12)	0.0171 (9)	−0.0130 (10)	0.0058 (8)	−0.0007 (8)
C22	0.0741 (19)	0.0377 (14)	0.0299 (12)	−0.0152 (14)	0.0015 (11)	0.0070 (10)
C23	0.0488 (14)	0.0380 (13)	0.0252 (10)	−0.0022 (11)	0.0021 (9)	0.0061 (9)
C1A	0.0416 (12)	0.0319 (12)	0.0231 (9)	0.0003 (10)	0.0055 (8)	0.0001 (8)
C2A	0.0558 (15)	0.0428 (14)	0.0249 (10)	−0.0114 (12)	−0.0013 (9)	0.0040 (9)
C3A	0.077 (2)	0.0572 (18)	0.0314 (12)	−0.0266 (16)	−0.0046 (12)	0.0011 (11)
C4A	0.104 (2)	0.0472 (17)	0.0410 (14)	−0.0374 (17)	0.0040 (14)	−0.0005 (12)
C5A	0.092 (2)	0.0407 (15)	0.0342 (13)	−0.0140 (15)	0.0065 (13)	0.0072 (11)
C6A	0.0557 (15)	0.0338 (13)	0.0240 (10)	−0.0039 (11)	0.0043 (9)	0.0022 (9)
C1B	0.0363 (11)	0.0294 (11)	0.0202 (9)	0.0032 (9)	−0.0011 (8)	0.0016 (8)
C2B	0.0401 (13)	0.0606 (17)	0.0287 (11)	0.0152 (12)	−0.0010 (9)	−0.0082 (11)
C3B	0.0399 (14)	0.081 (2)	0.0400 (13)	0.0209 (14)	−0.0074 (10)	−0.0121 (13)
C4B	0.0489 (15)	0.0501 (16)	0.0305 (12)	0.0024 (13)	−0.0105 (10)	−0.0006 (10)
C5B	0.0552 (15)	0.0591 (17)	0.0191 (10)	−0.0084 (13)	0.0007 (9)	0.0023 (10)
C6B	0.0401 (13)	0.0564 (16)	0.0220 (10)	0.0003 (12)	0.0042 (8)	0.0013 (10)
C1C	0.0318 (10)	0.0367 (12)	0.0180 (9)	0.0049 (9)	0.0066 (7)	0.0008 (8)
C2C	0.0412 (12)	0.0409 (14)	0.0296 (10)	0.0073 (11)	0.0138 (9)	−0.0022 (9)
C3C	0.0614 (17)	0.0448 (15)	0.0342 (12)	0.0128 (13)	0.0175 (11)	−0.0029 (10)
C4C	0.0507 (16)	0.071 (2)	0.0425 (14)	0.0266 (15)	0.0195 (11)	−0.0012 (13)
C5C	0.0335 (14)	0.090 (3)	0.0632 (17)	0.0040 (15)	0.0216 (12)	−0.0104 (16)
C6C	0.0374 (13)	0.0560 (17)	0.0429 (13)	−0.0036 (12)	0.0139 (10)	−0.0054 (11)
C1D	0.0412 (12)	0.0432 (14)	0.0174 (9)	−0.0092 (10)	0.0088 (8)	−0.0040 (8)
C2D	0.0445 (13)	0.0522 (15)	0.0243 (10)	−0.0115 (12)	0.0070 (9)	0.0041 (10)
C3D	0.0653 (17)	0.0568 (17)	0.0289 (11)	−0.0141 (15)	0.0158 (11)	0.0059 (11)
C4D	0.0581 (17)	0.070 (2)	0.0386 (13)	−0.0245 (15)	0.0250 (11)	−0.0068 (12)
C5D	0.0507 (16)	0.066 (2)	0.0603 (17)	−0.0054 (15)	0.0290 (13)	−0.0093 (15)
C6D	0.0494 (15)	0.0484 (16)	0.0423 (13)	−0.0043 (13)	0.0193 (11)	−0.0062 (11)
C1E	0.0427 (12)	0.0358 (13)	0.0198 (9)	−0.0067 (10)	0.0015 (8)	−0.0032 (8)
C2E	0.0484 (14)	0.0616 (17)	0.0230 (10)	−0.0045 (13)	0.0049 (9)	−0.0039 (10)
C3E	0.0586 (16)	0.0666 (19)	0.0203 (10)	−0.0040 (15)	−0.0013 (10)	−0.0051 (10)
C4E	0.0530 (15)	0.0497 (16)	0.0278 (11)	−0.0079 (13)	−0.0098 (10)	−0.0034 (10)
C5E	0.0435 (13)	0.0538 (16)	0.0327 (12)	−0.0080 (12)	−0.0021 (10)	0.0043 (11)
C6E	0.0448 (13)	0.0431 (14)	0.0227 (10)	−0.0098 (11)	0.0018 (9)	0.0014 (9)
C1F	0.0431 (13)	0.0376 (13)	0.0242 (10)	−0.0076 (10)	0.0059 (8)	−0.0015 (9)
C2F	0.0463 (13)	0.0431 (14)	0.0248 (10)	−0.0099 (11)	0.0084 (9)	−0.0027 (9)
C3F	0.0661 (17)	0.0425 (15)	0.0375 (13)	−0.0076 (13)	0.0171 (11)	−0.0096 (11)
C4F	0.078 (2)	0.0376 (15)	0.0483 (15)	0.0073 (14)	0.0135 (13)	0.0001 (12)
C5F	0.088 (2)	0.0457 (17)	0.0427 (15)	0.0098 (16)	−0.0065 (14)	0.0002 (12)
C6F	0.0735 (18)	0.0430 (16)	0.0313 (12)	0.0033 (14)	−0.0045 (11)	−0.0044 (10)
N1S	0.084 (3)	0.040 (3)	0.098 (4)	0.014 (3)	0.049 (3)	−0.006 (2)
C1S	0.089 (3)	0.046 (3)	0.101 (4)	0.015 (3)	0.051 (3)	−0.002 (2)
C2S	0.098 (3)	0.055 (3)	0.105 (4)	0.016 (3)	0.051 (3)	0.004 (2)
N1T	0.113 (4)	0.071 (4)	0.119 (5)	0.021 (4)	0.033 (4)	−0.004 (3)
C1T	0.109 (4)	0.069 (4)	0.118 (4)	0.020 (3)	0.037 (4)	0.000 (3)
C2T	0.104 (4)	0.065 (3)	0.114 (4)	0.017 (3)	0.044 (3)	0.004 (3)

### \ Bis(µ-1H-imidazole-2-thiolato)-κ2N3:S;\ κ2S:N3-bis[cyanido(triphenylphosphine-κP)\ palladium(II)] acetonitrile 0.58-solvate (2) Geometric parameters (Å, º)

**Table:**

Pd1—C1	1.957 (2)	C6B—H6BA	0.9500
Pd1—N11	2.0346 (17)	C1C—C6C	1.374 (3)
Pd1—P1	2.2914 (5)	C1C—C2C	1.394 (3)
Pd1—S2	2.3541 (5)	C2C—C3C	1.394 (3)
Pd2—C2	1.943 (2)	C2C—H2CA	0.9500
Pd2—N21	2.0345 (17)	C3C—C4C	1.366 (3)
Pd2—P2	2.2984 (5)	C3C—H3CA	0.9500
Pd2—S1	2.3542 (5)	C4C—C5C	1.373 (4)
S1—C11	1.734 (2)	C4C—H4CA	0.9500
S2—C21	1.733 (2)	C5C—C6C	1.383 (3)
P1—C1A	1.813 (2)	C5C—H5CA	0.9500
P1—C1C	1.823 (2)	C6C—H6CA	0.9500
P1—C1B	1.8247 (18)	C1D—C6D	1.373 (3)
P2—C1F	1.815 (2)	C1D—C2D	1.400 (3)
P2—C1D	1.820 (2)	C2D—C3D	1.384 (3)
P2—C1E	1.830 (2)	C2D—H2DA	0.9500
N1—C1	1.143 (3)	C3D—C4D	1.369 (4)
N2—C2	1.148 (3)	C3D—H3DA	0.9500
N11—C11	1.333 (2)	C4D—C5D	1.381 (4)
N11—C13	1.382 (3)	C4D—H4DA	0.9500
N12—C11	1.350 (3)	C5D—C6D	1.395 (3)
N12—C12	1.367 (3)	C5D—H5DA	0.9500
N12—H12A	0.8800	C6D—H6DA	0.9500
N21—C21	1.337 (3)	C1E—C6E	1.385 (3)
N21—C23	1.377 (3)	C1E—C2E	1.390 (3)
N22—C21	1.348 (3)	C2E—C3E	1.394 (3)
N22—C22	1.365 (3)	C2E—H2EA	0.9500
N22—H22A	0.8800	C3E—C4E	1.363 (3)
C12—C13	1.349 (3)	C3E—H3EA	0.9500
C12—H13A	0.9500	C4E—C5E	1.383 (3)
C13—H14A	0.9500	C4E—H4EA	0.9500
C22—C23	1.348 (3)	C5E—C6E	1.388 (3)
C22—H23A	0.9500	C5E—H5EA	0.9500
C23—H24A	0.9500	C6E—H6EA	0.9500
C1A—C6A	1.392 (3)	C1F—C6F	1.393 (3)
C1A—C2A	1.400 (3)	C1F—C2F	1.395 (3)
C2A—C3A	1.381 (3)	C2F—C3F	1.380 (3)
C2A—H2AA	0.9500	C2F—H2FA	0.9500
C3A—C4A	1.384 (3)	C3F—C4F	1.385 (3)
C3A—H3AA	0.9500	C3F—H3FA	0.9500
C4A—C5A	1.395 (3)	C4F—C5F	1.379 (3)
C4A—H4AA	0.9500	C4F—H4FA	0.9500
C5A—C6A	1.384 (3)	C5F—C6F	1.379 (4)
C5A—H5AA	0.9500	C5F—H5FA	0.9500
C6A—H6AA	0.9500	C6F—H6FA	0.9500
C1B—C6B	1.383 (3)	N1S—C1S	1.163 (10)
C1B—C2B	1.391 (3)	C1S—C2S	1.405 (11)
C2B—C3B	1.387 (3)	C2S—H2S1	0.9800
C2B—H2BA	0.9500	C2S—H2S2	0.9800
C3B—C4B	1.374 (3)	C2S—H2S3	0.9800
C3B—H3BA	0.9500	N1T—C1T	1.167 (10)
C4B—C5B	1.364 (3)	C1T—C2T	1.405 (11)
C4B—H4BA	0.9500	C2T—H2T1	0.9800
C5B—C6B	1.387 (3)	C2T—H2T2	0.9800
C5B—H5BA	0.9500	C2T—H2T3	0.9800
			
C1—Pd1—N11	179.31 (8)	C6B—C5B—H5BA	120.1
C1—Pd1—P1	87.19 (6)	C1B—C6B—C5B	120.9 (2)
N11—Pd1—P1	92.80 (4)	C1B—C6B—H6BA	119.6
C1—Pd1—S2	87.99 (6)	C5B—C6B—H6BA	119.6
N11—Pd1—S2	92.06 (5)	C6C—C1C—C2C	118.4 (2)
P1—Pd1—S2	173.855 (19)	C6C—C1C—P1	122.32 (18)
C2—Pd2—N21	178.38 (8)	C2C—C1C—P1	119.14 (16)
C2—Pd2—P2	89.18 (7)	C3C—C2C—C1C	120.2 (2)
N21—Pd2—P2	92.44 (5)	C3C—C2C—H2CA	119.9
C2—Pd2—S1	86.54 (7)	C1C—C2C—H2CA	119.9
N21—Pd2—S1	91.84 (5)	C4C—C3C—C2C	120.2 (2)
P2—Pd2—S1	174.489 (19)	C4C—C3C—H3CA	119.9
C11—S1—Pd2	103.47 (7)	C2C—C3C—H3CA	119.9
C21—S2—Pd1	103.09 (7)	C3C—C4C—C5C	120.0 (2)
C1A—P1—C1C	105.05 (10)	C3C—C4C—H4CA	120.0
C1A—P1—C1B	106.83 (9)	C5C—C4C—H4CA	120.0
C1C—P1—C1B	103.83 (9)	C4C—C5C—C6C	120.1 (3)
C1A—P1—Pd1	112.97 (6)	C4C—C5C—H5CA	120.0
C1C—P1—Pd1	115.90 (6)	C6C—C5C—H5CA	120.0
C1B—P1—Pd1	111.44 (7)	C1C—C6C—C5C	121.2 (2)
C1F—P2—C1D	104.60 (11)	C1C—C6C—H6CA	119.4
C1F—P2—C1E	105.46 (10)	C5C—C6C—H6CA	119.4
C1D—P2—C1E	104.41 (9)	C6D—C1D—C2D	119.2 (2)
C1F—P2—Pd2	114.49 (7)	C6D—C1D—P2	121.09 (18)
C1D—P2—Pd2	113.82 (7)	C2D—C1D—P2	119.39 (18)
C1E—P2—Pd2	113.06 (7)	C3D—C2D—C1D	119.9 (2)
C11—N11—C13	107.43 (18)	C3D—C2D—H2DA	120.0
C11—N11—Pd1	122.63 (14)	C1D—C2D—H2DA	120.0
C13—N11—Pd1	129.94 (15)	C4D—C3D—C2D	120.7 (3)
C11—N12—C12	108.74 (19)	C4D—C3D—H3DA	119.7
C11—N12—H12A	125.6	C2D—C3D—H3DA	119.7
C12—N12—H12A	125.6	C3D—C4D—C5D	119.7 (3)
C21—N21—C23	107.19 (18)	C3D—C4D—H4DA	120.1
C21—N21—Pd2	122.77 (15)	C5D—C4D—H4DA	120.1
C23—N21—Pd2	130.03 (15)	C4D—C5D—C6D	120.2 (3)
C21—N22—C22	108.90 (19)	C4D—C5D—H5DA	119.9
C21—N22—H22A	125.6	C6D—C5D—H5DA	119.9
C22—N22—H22A	125.6	C1D—C6D—C5D	120.3 (3)
N1—C1—Pd1	178.1 (2)	C1D—C6D—H6DA	119.8
N2—C2—Pd2	178.2 (2)	C5D—C6D—H6DA	119.8
N11—C11—N12	108.68 (19)	C6E—C1E—C2E	119.17 (18)
N11—C11—S1	125.60 (16)	C6E—C1E—P2	120.21 (14)
N12—C11—S1	125.72 (16)	C2E—C1E—P2	120.60 (17)
C13—C12—N12	106.7 (2)	C1E—C2E—C3E	120.1 (2)
C13—C12—H13A	126.6	C1E—C2E—H2EA	119.9
N12—C12—H13A	126.6	C3E—C2E—H2EA	119.9
C12—C13—N11	108.4 (2)	C4E—C3E—C2E	120.3 (2)
C12—C13—H14A	125.8	C4E—C3E—H3EA	119.9
N11—C13—H14A	125.8	C2E—C3E—H3EA	119.9
N21—C21—N22	108.60 (19)	C3E—C4E—C5E	120.2 (2)
N21—C21—S2	126.07 (16)	C3E—C4E—H4EA	119.9
N22—C21—S2	125.32 (17)	C5E—C4E—H4EA	119.9
C23—C22—N22	106.5 (2)	C4E—C5E—C6E	120.1 (2)
C23—C22—H23A	126.8	C4E—C5E—H5EA	119.9
N22—C22—H23A	126.8	C6E—C5E—H5EA	119.9
C22—C23—N21	108.8 (2)	C1E—C6E—C5E	120.17 (19)
C22—C23—H24A	125.6	C1E—C6E—H6EA	119.9
N21—C23—H24A	125.6	C5E—C6E—H6EA	119.9
C6A—C1A—C2A	119.2 (2)	C6F—C1F—C2F	118.1 (2)
C6A—C1A—P1	123.45 (16)	C6F—C1F—P2	118.58 (17)
C2A—C1A—P1	117.38 (16)	C2F—C1F—P2	123.18 (16)
C3A—C2A—C1A	120.7 (2)	C3F—C2F—C1F	120.8 (2)
C3A—C2A—H2AA	119.7	C3F—C2F—H2FA	119.6
C1A—C2A—H2AA	119.7	C1F—C2F—H2FA	119.6
C2A—C3A—C4A	119.9 (2)	C2F—C3F—C4F	120.3 (2)
C2A—C3A—H3AA	120.0	C2F—C3F—H3FA	119.8
C4A—C3A—H3AA	120.0	C4F—C3F—H3FA	119.8
C3A—C4A—C5A	119.8 (2)	C5F—C4F—C3F	119.4 (3)
C3A—C4A—H4AA	120.1	C5F—C4F—H4FA	120.3
C5A—C4A—H4AA	120.1	C3F—C4F—H4FA	120.3
C6A—C5A—C4A	120.4 (2)	C4F—C5F—C6F	120.5 (2)
C6A—C5A—H5AA	119.8	C4F—C5F—H5FA	119.7
C4A—C5A—H5AA	119.8	C6F—C5F—H5FA	119.7
C5A—C6A—C1A	120.0 (2)	C5F—C6F—C1F	120.8 (2)
C5A—C6A—H6AA	120.0	C5F—C6F—H6FA	119.6
C1A—C6A—H6AA	120.0	C1F—C6F—H6FA	119.6
C6B—C1B—C2B	118.82 (17)	N1S—C1S—C2S	176.2 (11)
C6B—C1B—P1	121.48 (16)	C1S—C2S—H2S1	109.5
C2B—C1B—P1	119.70 (14)	C1S—C2S—H2S2	109.5
C3B—C2B—C1B	119.6 (2)	H2S1—C2S—H2S2	109.5
C3B—C2B—H2BA	120.2	C1S—C2S—H2S3	109.5
C1B—C2B—H2BA	120.2	H2S1—C2S—H2S3	109.5
C4B—C3B—C2B	120.7 (2)	H2S2—C2S—H2S3	109.5
C4B—C3B—H3BA	119.7	N1T—C1T—C2T	167.3 (16)
C2B—C3B—H3BA	119.7	C1T—C2T—H2T1	109.5
C5B—C4B—C3B	120.1 (2)	C1T—C2T—H2T2	109.5
C5B—C4B—H4BA	119.9	H2T1—C2T—H2T2	109.5
C3B—C4B—H4BA	119.9	C1T—C2T—H2T3	109.5
C4B—C5B—C6B	119.8 (2)	H2T1—C2T—H2T3	109.5
C4B—C5B—H5BA	120.1	H2T2—C2T—H2T3	109.5
			
C13—N11—C11—N12	−1.0 (2)	Pd1—P1—C1C—C6C	107.61 (17)
Pd1—N11—C11—N12	179.69 (12)	C1A—P1—C1C—C2C	166.82 (15)
C13—N11—C11—S1	178.50 (14)	C1B—P1—C1C—C2C	54.80 (17)
Pd1—N11—C11—S1	−0.8 (2)	Pd1—P1—C1C—C2C	−67.74 (16)
C12—N12—C11—N11	0.5 (2)	C6C—C1C—C2C—C3C	−0.1 (3)
C12—N12—C11—S1	−179.08 (15)	P1—C1C—C2C—C3C	175.43 (15)
Pd2—S1—C11—N11	−58.55 (17)	C1C—C2C—C3C—C4C	0.1 (3)
Pd2—S1—C11—N12	120.92 (16)	C2C—C3C—C4C—C5C	0.7 (4)
C11—N12—C12—C13	0.3 (2)	C3C—C4C—C5C—C6C	−1.5 (4)
N12—C12—C13—N11	−1.0 (2)	C2C—C1C—C6C—C5C	−0.7 (3)
C11—N11—C13—C12	1.3 (2)	P1—C1C—C6C—C5C	−176.07 (19)
Pd1—N11—C13—C12	−179.55 (14)	C4C—C5C—C6C—C1C	1.5 (4)
C23—N21—C21—N22	−1.0 (2)	C1F—P2—C1D—C6D	−23.41 (19)
Pd2—N21—C21—N22	179.80 (12)	C1E—P2—C1D—C6D	−133.98 (18)
C23—N21—C21—S2	177.87 (14)	Pd2—P2—C1D—C6D	102.27 (17)
Pd2—N21—C21—S2	−1.3 (2)	C1F—P2—C1D—C2D	163.28 (16)
C22—N22—C21—N21	0.7 (2)	C1E—P2—C1D—C2D	52.72 (18)
C22—N22—C21—S2	−178.16 (16)	Pd2—P2—C1D—C2D	−71.03 (17)
Pd1—S2—C21—N21	−57.77 (17)	C6D—C1D—C2D—C3D	−0.8 (3)
Pd1—S2—C21—N22	120.92 (16)	P2—C1D—C2D—C3D	172.62 (16)
C21—N22—C22—C23	−0.1 (2)	C1D—C2D—C3D—C4D	0.4 (3)
N22—C22—C23—N21	−0.5 (2)	C2D—C3D—C4D—C5D	0.8 (4)
C21—N21—C23—C22	0.9 (2)	C3D—C4D—C5D—C6D	−1.6 (4)
Pd2—N21—C23—C22	−179.96 (14)	C2D—C1D—C6D—C5D	0.0 (3)
C1C—P1—C1A—C6A	−98.1 (2)	P2—C1D—C6D—C5D	−173.35 (18)
C1B—P1—C1A—C6A	11.7 (2)	C4D—C5D—C6D—C1D	1.2 (4)
Pd1—P1—C1A—C6A	134.63 (18)	C1F—P2—C1E—C6E	96.5 (2)
C1C—P1—C1A—C2A	82.64 (19)	C1D—P2—C1E—C6E	−153.57 (19)
C1B—P1—C1A—C2A	−167.49 (17)	Pd2—P2—C1E—C6E	−29.3 (2)
Pd1—P1—C1A—C2A	−44.6 (2)	C1F—P2—C1E—C2E	−81.6 (2)
C6A—C1A—C2A—C3A	2.1 (4)	C1D—P2—C1E—C2E	28.4 (2)
P1—C1A—C2A—C3A	−178.7 (2)	Pd2—P2—C1E—C2E	152.62 (17)
C1A—C2A—C3A—C4A	−1.2 (4)	C6E—C1E—C2E—C3E	−0.1 (4)
C2A—C3A—C4A—C5A	−0.7 (5)	P2—C1E—C2E—C3E	178.0 (2)
C3A—C4A—C5A—C6A	1.8 (5)	C1E—C2E—C3E—C4E	−0.3 (4)
C4A—C5A—C6A—C1A	−0.9 (4)	C2E—C3E—C4E—C5E	0.5 (4)
C2A—C1A—C6A—C5A	−1.0 (4)	C3E—C4E—C5E—C6E	−0.4 (4)
P1—C1A—C6A—C5A	179.8 (2)	C2E—C1E—C6E—C5E	0.2 (3)
C1A—P1—C1B—C6B	−83.0 (2)	P2—C1E—C6E—C5E	−177.91 (18)
C1C—P1—C1B—C6B	27.7 (2)	C4E—C5E—C6E—C1E	0.1 (4)
Pd1—P1—C1B—C6B	153.15 (17)	C1D—P2—C1F—C6F	90.2 (2)
C1A—P1—C1B—C2B	97.0 (2)	C1E—P2—C1F—C6F	−160.00 (19)
C1C—P1—C1B—C2B	−152.31 (19)	Pd2—P2—C1F—C6F	−35.1 (2)
Pd1—P1—C1B—C2B	−26.9 (2)	C1D—P2—C1F—C2F	−85.8 (2)
C6B—C1B—C2B—C3B	0.4 (4)	C1E—P2—C1F—C2F	24.1 (2)
P1—C1B—C2B—C3B	−179.6 (2)	Pd2—P2—C1F—C2F	148.98 (17)
C1B—C2B—C3B—C4B	−0.9 (4)	C6F—C1F—C2F—C3F	0.0 (4)
C2B—C3B—C4B—C5B	0.6 (4)	P2—C1F—C2F—C3F	175.98 (19)
C3B—C4B—C5B—C6B	0.2 (4)	C1F—C2F—C3F—C4F	−1.4 (4)
C2B—C1B—C6B—C5B	0.4 (4)	C2F—C3F—C4F—C5F	1.6 (4)
P1—C1B—C6B—C5B	−179.63 (19)	C3F—C4F—C5F—C6F	−0.5 (5)
C4B—C5B—C6B—C1B	−0.7 (4)	C4F—C5F—C6F—C1F	−0.8 (5)
C1A—P1—C1C—C6C	−17.83 (19)	C2F—C1F—C6F—C5F	1.1 (4)
C1B—P1—C1C—C6C	−129.86 (18)	P2—C1F—C6F—C5F	−175.1 (2)

### \ Bis(µ-1H-imidazole-2-thiolato)-κ2N3:S;\ κ2S:N3-bis[cyanido(triphenylphosphine-κP)\ palladium(II)] acetonitrile 0.58-solvate (2) Hydrogen-bond geometry (Å, º)

**Table:**

*D*—H···*A*	*D*—H	H···*A*	*D*···*A*	*D*—H···*A*
N12—H12*A*···N2^i^	0.88	1.90	2.770 (3)	169
N22—H22*A*···N1^ii^	0.88	1.92	2.760 (3)	160
C12—H13*A*···N1*S*	0.95	2.35	3.261 (7)	161
C22—H23*A*···N1*T*	0.95	2.29	3.081 (12)	141

Symmetry codes: (i) −*x*, *y*+1/2, −*z*+1/2; (ii) −*x*+1, *y*+1/2, −*z*+1/2.
